# Superior success rate of intracavitary electrocardiogram guidance for peripherally inserted central catheter placement in patients with cancer: A randomized open-label controlled multicenter study

**DOI:** 10.1371/journal.pone.0171630

**Published:** 2017-03-09

**Authors:** Ling Yuan, Rongmei Li, Aifeng Meng, Yuling Feng, Xiancui Wu, Yiqun Yang, Ping Chen, Zhenzhu Qiu, Jing Qi, Chuanying Chen, Jia Wei, Minyi Qin, Weiwei Kong, Xiangyu Chen, Wei Xu

**Affiliations:** 1 The Comprehensive Cancer Centre of Drum Tower Hospital, Medical School of Nanjing University, Clinical Cancer Institute of Nanjing University, Nanjing, Jiangsu Province, China; 2 Department of Nursing, Jiangsu Cancer Hospital, Nanjing, Jiangsu Province, China; 3 The Comprehensive Cancer Centre of People’s Hospital Affiliated to Jiangsu University, Zhenjiang, Jiangsu Province, China; 4 Department of Medical Oncology, Nanjing Hospital Affiliated to Nanjing Medical University, Nanjing, Jiangsu Province, China; 5 Department of Nursing, The First Affiliated Hospital of Soochow University, Suzhou, Jiangsu Province, China; 6 Department of Medical Oncology, The Second Affiliated Hospital of Nanjing Medical University, Nanjing, Jiangsu Province, China; 7 Department of Medical Oncology, The Second Affiliated Hospital of Soochow University, Suzhou, Jiangsu Province, China; 8 Department of Medical Oncology, Nanjing Jinling Hospital, Nanjing, Jiangsu Province, China; 9 Department of Medical Imaging, Drum Tower Hospital, Medical School of Nanjing University, Nanjing, Jiangsu Province, China; 10 Department of Nursing, Medical School of Nanjing University, Clinical Cancer Institute of Nanjing University, Nanjing, Jiangsu Province, China; 11 Department of Cardiology, Drum Tower Hospital, Medical School of Nanjing University, Nanjing, Jiangsu Province, China; Public Library of Science, FRANCE

## Abstract

**Background:**

Intracavitary electrocardiogram (IC ECG) guidance emerges as a new technique for peripherally inserted central catheters (PICCs) placement and demonstrates many potential advantages in recent observational studies.

**Aims:**

To determine whether IC ECG-guided PICCs provide more accurate positioning of catheter tips compared to conventional anatomical landmarks in patients with cancer undergoing chemotherapy.

**Methods:**

In this multicenter, open-label, randomized controlled study (ClinicalTrials.gov number, NCT02409589), a total of 1,007 adult patients were assigned to receive either IC ECG guidance (n = 500) or anatomical landmark guidance (n = 507) for PICC positioning. The confirmative catheter tip positioning x-ray data were centrally interpreted by independent radiologists. All reported analyses in the overall population were performed on an intention-to-treat basis. Analyses of pre-specified subgroups and a selected large subpopulation were conducted to explore consistency and accuracy.

**Results:**

In the IC ECG-guided group, the first-attempt success rate was 89.2% (95% confidence interval [CI], 86.5% to 91.9%), which was significantly higher than 77.4% (95% CI, 73.7% to 81.0%) in the anatomical landmark group (P < 0.0001). This trend of superiority of IC ECG guidance was consistently noted in almost all prespecified patient subgroups and two selected large subpopulations, even when using optimal target rates for measurement. In contrast, the superiority nearly disappeared when PICCs were used via the left instead of right arms (interaction P-value = 0.021). No catheter-related adverse events were reported during the PICC intra-procedures in either group.

**Conclusions:**

Our findings indicated that the IC ECG-guided method had a more favorable positioning accuracy versus traditional anatomical landmarks for PICC placement in adult patients with cancer undergoing chemotherapy. Furthermore, there were no significant safety concerns reported for catheterization using the two techniques.

## Introduction

Chemotherapy as a routine treatment option for patients with cancer is usually administrated intravenously using a peripherally inserted central catheter (PICC) [1–5]. Since catheters placed outside of the cavo-atrial junction (CAJ) are potentially associated with higher risk of thrombosis and infection [1–3], it is essential that the catheter tip is in an ideal position when PICCs used for chemotherapy are left in place for several weeks or months [6, 7]. Most recommendations from the USA [8–10] propose that the tip should be placed in the lower third of the superior vena cava (SVC), while European guidelines [11, 12] often regard the upper part of the atrium as acceptable. Specifically, Pittiruti et al. [13, 14] agreed that for chemotherapy, the tip should preferably be placed in the lower third of the SVC. Chinese nursing practice standards require that the catheter tip be placed in the SVC [15]. Since there is no method for assessing tip positioning that can exclude misplacement with absolute certainty [16], any new method with high accuracy at first-attempt insertion warrants research.

The conventional anatomical landmark method exploits the estimated length from the puncture site to the junction of the SVC/right atrium (SVC-RA) to guide tip placement. This method is associated with relatively lower financial costs and acceptable performance, and has become the first choice for patients with poor economic status and good vascular access in China [5]. Nevertheless, additional post-procedural chest X-rays (exposure of patients to radiation and extra cost) are required later to confirm central line placement. Following on from this simple technique, there is a growing body of evidence from around the world regarding the use of intracavitary electrocardiography (IC ECG) to guide PICC placement. The current findings indicate that this alterative technique could be as accurate as radiological methods as well as safe, while providing real-time monitoring and less need for X-ray confirmation [1, 13]. In a recent prospective, non-controlled Italian study, Rossetti et al. reported that IC ECG was up to 95.8% as effective as X-ray [14]. However, there is a lack of randomized controlled studies comparing these two methods properly. Therefore, the current study tested the hypothesis of whether IC ECG-guided technique is more accurate than landmark technique for PICC placement in patients with cancer undergoing chemotherapy. We investigated how well the two techniques perform among patient subgroups. This study provides substantial initial Chinese patient data with which to improve PICC technique for local use.

## Materials and methods

### Study design

This was a multicenter, open-label, randomized controlled (EGG) study of IC ECG guidance versus anatomical landmark guidance in patients undergoing chemotherapy for different cancers (NCT02409589; study protocol [S1 and S2 Files] and Consolidated Standards of Reporting Trials (CONSORT) checklist available in S3 File). There was a 1-week delay in making public the online registry of this study because our study team had to develop the information used for the registry in English. The authors confirm that all ongoing and related trials for this IC ECG intervention are registered. Eligible patients enrolled by sites obtained a unique patient number and were randomly assigned to undergo IC ECG guidance or anatomical landmark guidance for PICC insertion in a 1:1 ratio. A stratified block randomization with randomly varying block sizes of 2 and 4 was performed, and stratification by site was used. Random assignment was performed by a statistician from Shanghai Knowlands MedPharm Consulting Co., Ltd and implemented via random envelopes assigned to each site. Sequences were concealed from patients and clinical staff until assignment. The study protocol was centrally approved by the independent Medical Ethics Committee (IEC) of Drum Tower Hospital, Medical School of Nanjing University on March 10, 2015.

### Patients

The study was conducted at eight centers in China. Eligible inpatients or outpatients were aged 18 to 80 years, had malignant tumors that required the periodical infusion of chemotherapy drugs via three-valve PICCs, and had normal P-waves according to surface ECG recordings prior to PICC insertion. Patients also had to provide signed informed consent prior to enrollment in the study.

Patients with the following conditions were excluded: heart diseases such as valvular heart disease, atrial fibrillation, supraventricular tachycardia, pulmonary heart disease, or the presence of a pacemaker and past history of cardiac surgery that may affect P-waves, or the inability to lie in the prostrate or semi-supine position.

### PICC methods

Eligible patients underwent traditional anatomical landmark or IC ECG-guided technique for PICC insertion. We used Rountree’s method [17] to measure catheter insertion length. A single-lumen PICC kit (Groshong NXT ClearVue, 4 F) manufactured by Bard Access Systems, Inc. (Utah, USA) and a C100 specialized cardiovascular monitor from Shenzhen Comen Medical Instruments Co., Ltd. were used during catheterization. In the landmark group, if we suspected that the catheter had been inserted outside the SVC, an ultrasound examination of the subclavian vein, internal jugular vein and external jugular vein was performed to detect and adjust any malposition of the catheter.

In the IC ECG group, we used a column of saline contained in the catheter as an intracavitary (endovascular) electrode [13]. IC ECG was performed to guide the positioning of the PICC tip while using an intravenous gravity drip of normal saline to form the column [18]. The new IC ECG guidance system used in the study had been offered a National Utility Model Patent (No.: ZL-2014-2-0436176.7) previously. The same puncture procedure was applied. In accordance with our pilot study, after puncture was performed, the catheter was advanced gently with sequential monitoring of ECG P-wave shape and amplitude by a bedside ECG monitor as follows: (1) when the catheter tip was located outside of the SVC or was just entering the SVC, no obvious change in P-wave occurred relative to surface ECG; (2) when continuing to slowly advance the catheter along the SVC, the P-wave would enlarge gradually; (3) when the catheter tip reached the atrium, the following changes in P-wave were observed: the low-frequency wave shifted to high frequency and the shape changed from obtuse to very sharp with either the PR segment moving down or negative P-wave starting to appear (amplitude ≥ 1 mm). The catheter was immediately pulled back slowly until the P-wave returned to a low-frequency obtuse shape with no negative wave, which then indicated that the catheter tip was located in the lower third of the SVC or at the CAJ (S1 Fig). Therefore, no ultrasound examinations were required to adjust PICC placement in this group.

### Efficacy evaluation

The primary analysis was an intention-to-treat (ITT) assessment of first-attempt success rates with IC ECG guidance compared with anatomical landmark guidance for PICC insertion. The first-attempt success rate as a primary efficacy endpoint was defined as the proportion of patients whose catheter tip was in the SVC or at the CAJ during the first attempt of PICC insertion as confirmed by chest X-ray. If the catheter tip was in the lower third of the SVC or at the CAJ, the placement was regarded as optimal. Furthermore, the proportion of patients whose catheter tip resided in the RA space at the first attempt was also summarized. In the study, we utilized the reported methodology and radiologic criteria [19] for the post-procedural review of the tip position. When the PICC tip was positioned in the right atrium, the distance between the cavo–atrial junction and the tip was determined. Then the right atrium is divided into three equal segments of upper 1/3, middle 1/3 and lower 1/3. The confirmative X-ray data in the study were interpreted separately by two independent radiologists. If inconsistent findings were noted, a third radiologist would further check the X-ray recordings and make a decision, and the majority decision was taken as the final result. In total, the third radiologist checked 43 X-ray recordings until the determination of primary endpoint. All of the independent radiologists are always blinded to treatment assignment. In addition to demographic and medical condition data, physical and laboratory examinations were performed prior to the start of and/or during PICC insertion.

### Statistical analysis

All randomly assigned patients were included as an ITT population in the primary analysis. No missing data were imputed and the as-observed rule was implemented. The primary efficacy parameters of first-attempt success rates were compared using a Pearson’s chi-squared test with a two-sided 5% significance level; 95% confidence intervals (CIs) of the success rate and rate difference were provided to indicate precision. The CIs used normal approximation or the Clopper-Pearson’s exact method. Interactions between planned covariates and PICC techniques were evaluated using a logistic model. The P-values for interaction were reported. The prespecified stratifications were based on the following covariates: gender, age group, body mass index (BMI) classification, cancer duration since diagnosis, current cancer metastasis status, prior chemotherapy, prior chest radiotherapy, smoking status, current activity level, prior CVCs, prior PICC insertion, arm side of PICC, and body posture during PICC insertion. Additional analyses of selected subpopulations were performed. BMI classification was derived using Chinese criteria [20]. Lesser activity was defined as an amount less than that of daily living (activities performed for self-care such as feeding, bathing, dressing, grooming, etc.) and higher activity as more than that required for daily living (ability to work normally, perform physical exercise, etc.) [21].

Assuming a first-attempt success rate of 88% for the anatomical landmark method as reported in previous studies [22–24], a sample size of 135 patients per group was assumed to have 90% power to detect a 10% increase in rate at a 2-sided Type I error rate of 5%. With an estimated 10% dropout rate during the study, 300 patients were randomized. Given the intention and feasibility to perform subgroup analyses and exploratory analysis on common PICCs-related complications as well, the sample size was enlarged to 1,000 randomized patients (500 per group). Two-sided p-values of less than 0.05 were considered statistically significant. No multiplicity adjustment was considered for the subgroup analyses. All statistical analyses were performed using SAS software, version 9.2 (SAS Institute, NC, USA) and R, version 3.1.2 [25].

## Results

### Patient characteristics

**Fig 1:**
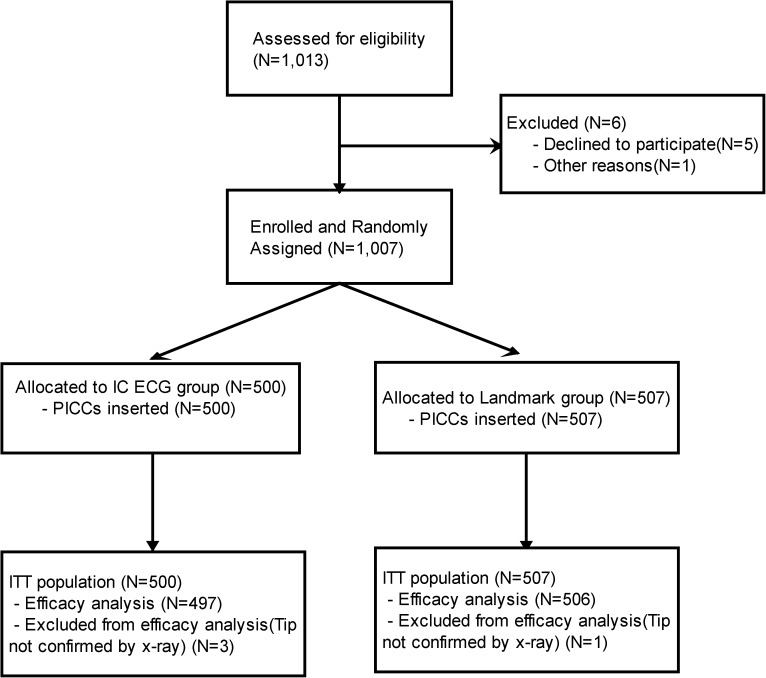
The Consolidated Standards of Reporting Trials (CONSORT) diagram depicting the trajectory of the trial. IC ECG: Intracavitary Electrocardiograph, ITT: Intention-To-Treat, PICCs: Peripherally Inserted Central Catheters.

Between late March and July 2015, a total of 1,007 patients were randomly assigned to receive either IC ECG guidance (n = 500) or anatomical landmark guidance (n = 507) for PICC positioning (ITT population; Fig 1). Four patients (IC ECG: n = 1; landmark: n = 3) refused to undergo X-ray confirmation for catheter tip positioning. No other protocol deviations occurred during the entire procedural period, although four patients were aged over 80.

Patient demographic and baseline disease characteristic data were well matched between the two study groups (Table 1 and S1 Table).

**Table 1:** Demographic details, smoking statuses, and activity levels of the study participants (ITT, N = 1,007).

	PICC method		All(N = 1,007)
	IC ECG(N = 500)	Landmark(N = 507)	
Age (years)			
Mean (SD)	57.4 (11.4)	58.3 (11.3)	57.9 (11.4)
18–65 years	363 (72.7%)	352 (69.6%)	715 (71.1%)
>65 years	136 (27.3%)	154 (30.4%)	290 (28.9%)
Gender (n [%])			
Male	247 (49.4%)	265 (52.6%)	512 (51.0%)
Female	253 (50.6%)	239 (47.4%)	492 (49.0%)
Body mass index (BMI, kg/m^2^)			
Mean (SD)	22.6 (3.4)	22.6 (3.1)	22.6 (3.3)
Weight classification* (n [%])			
Low weight (BMI < 18.5)	59 (11.8%)	45 (8.9%)	104 (10.4%)
Normal weight: BMI [18.5–24.0)	279 (55.9%)	292 (58.1%)	571 (57.0%)
Overweight: BMI [24.0–28.0)	131 (26.3%)	143 (28.4%)	274 (27.3%)
Obesity (BMI ≥ 28.0)	30 (6.0%)	23 (4.6%)	53 (5.3%)
Current activity amount (n [%])			
Less activity	28 (5.6%)	46 (9.1%)	74 (7.4%)
Moderate activity	103 (20.7%)	96 (18.9%)	199 (19.8%)
Large activity	366 (73.6%)	365 (72.0%)	731 (72.8%)
Smoking status (n [%])			
Never	278 (55.7%)	270 (53.3%)	548 (54.5%)
Former smoker	201 (40.3%)	225 (44.4%)	426 (42.3%)
Current smoker	20 (4.0%)	12 (2.4%)	32 (3.2%)

*Adult weight measures. The national health and family planning commission of the People's Republic of China. 2013-04-18. http://www.nhfpc.gov.cn/. Last accessed on 2015-Sep-2

BMI: Body Mass Index, IC ECG: Intracavitary Electrocardiograph, ITT: Intention-To-Treat, PICCs: Peripherally Inserted Central Catheters, SD: Standard Deviation.

### PICC insertion characteristics and safety

The PICC operating characteristics were comparable between the two techniques (S2 Table). Most PICCs were inserted via the right arm (77%), using the basilic vein (89%), and in the supine body posture (86%). For safety, intra-procedural heart rhythm, heart rate and blood oxygen levels were monitored in the IC ECG group, but no significant changes since the start of PICC insertion were noted (data not shown). No catheter-related adverse events or complications were reported during the PICC manipulation in either group.

### Accuracy of PICC tip positioning

During the PICC procedure, ultrasound examinations indicted that in 29 (5.8%) patients who underwent placement using the anatomical landmark technique, the PICC tip failed to reach the SVC area on the first attempt. Of them, eight patients experienced aberrant placement ≥ 3 times. Conversely, In the IC ECG guided group, tip misplacement was totally avoided due to real-time monitoring of changes in P-waves.

In our study, the first-attempt success rate in the IC ECG-guided group was up to 89.2% (95% CI, 86.5% to 91.9%), significantly higher than 77.4% (95% CI, 73.7% to 81.0%) in the anatomical landmark group (P < 0.0001, Table 2). A slightly less obvious superiority was observed with respect to optimal target rate or right atrium rate (Table 2). This trend of higher accuracy when using IC ECG guidance was consistently found in almost all of the prespecified patient subgroups (Table 3) and two selected large subpopulations (S3 and S4 Tables). However, the superiority of IC ECG guidance nearly disappeared when PICCs were inserted via the left arm, while it remained when the right side was used (interaction P-value = 0.021, Table 3).

**Table 2:** Catheter tip placement at first attempt and corresponding rates of outcome measures (ITT, N = 1,007).

	PICC Method		
	IC ECG(N = 500)	Landmark(N = 507)	p-value
Catheter tip positioning places at first attempt, n (%)			<0.0001
SVC upper 1/3	42 (8.4%)	42 (8.3%)	
SVC middle 1/3	82 (16.4%)	73 (14.5%)	
SVC lower 1/3	165 (33.1%)	108 (21.4%)	
SVC/RA junction	156 (31.3%)	167 (33.1%)	
RA upper 1/3	39 (7.8%)	63 (12.5%)	
RA middle 1/3	6 (1.2%)	20 (4.0%)	
RA lower 1/3	1 (0.2%)	2 (0.4%)	
Other places	8 (1.6%)	29 (5.8%)	
First-attempt target rate			<0.0001
n (%)	445(89.2%)	390(77.4%)	
Percent difference (95% CI)	11.8 (7.0 to16.6)	-	
Optimal target rate			0.0016
n (%)	321(64.3%)	275(54.6%)	
Percent difference (95% CI)	9.8 (3.5 to16.0)	-	
Right atrium rate			0.0003
n (%)	46(9.2%)	85(16.9%)	
Percent difference (95% CI)	-7.6 (-12.0 to -3.3)	-	

CI: Confidence Interval, IC ECG: Intracavitary Electrocardiograph, ITT: Intention-To-Treat, PICCs: Peripherally Inserted Central Catheters, SVC: Superior Vena Cava.

**Table 3:** First-attempt success rates with IC ECG versus landmark technique according to prespecified subgroups.

Subgroups	No.	PICC Method		Percent difference(95% CI)	p-value for interaction
		IC ECG N (%)	LandmarkN (%)		
Gender					0.841
Male	512	223 (90.3%)	211 (79.9%)	10.4 (3.9 to 16.8)	
Female	492	222 (88.1%)	177 (74.7%)	13.4 (6.2 to 20.6)	
Age group					0.718
18–65 years	715	319 (88.1%)	264 (75.6%)	12.5 (6.6 to 18.4)	
>65 years	290	126 (92.6)	126 (81.8)	10.8 (2.6 to 19.0)	
BMI classification*					0.690
<18.5	104	54 (91.5)	36 (80.0)	11.5 (-4.1 to 27.2)	
[18.5–24)	571	251 (90.3)	238 (81.5)	8.8 (2.8 to 14.8)	
[24–28)	274	113 (86.3)	101 (71.6)	14.6 (4.4 to 24.9)	
≥ 28	53	26 (86.7)	13 (56.5)	30.1 (6.5 to 53.8)	
Cancer duration					0.788
<1 month	454	207 (89.2)	168 (76.4)	12.9 (5.5 to 20.2)	
≥1 month	550	237 (89.4)	222 (78.4)	11.0 (4.6 to 17.4)	
Cancer metastasis					0.728
Yes	499	220 (89.1)	190 (76.0)	13.1 (6.5 to 19.6)	
No	496	220 (89.8)	198 (79.5)	10.3 (4.0 to 16.6)	
Prior chemotherapy					0.533
Present	268	119 (89.5)	108 (81.2)	8.3 (-0.9 to 17.5)	
Absent	736	323 (89.0)	282 (76.0)	13.0 (7.3 to 18.7)	
Prior chest radiotherapy					0.237
Present	103	46 (92.0)	37 (71.2)	20.8 (6.4 to 35.3)	
Absent	901	397 (88.8)	353 (78.3)	10.5 (5.5 to 15.6)	
Smoking					0.286
Never	548	245 (88.4)	198 (73.9)	14.6 (7.7 to 21.4)	
Former smoker	426	185 (92.0)	182 (81.3)	10.8 (4.0 to 17.6)	
Current smoker	32	15 (75.0)	10 (83.3)	-8.3 (-36.7 to 20.0)	
Current activity amount					0.520
Less	74	22 (78.6)	32 (71.1)	7.5 (-15.6 to 30.5)	
Moderate	199	93 (90.3)	71 (74.0)	16.3 (4.8 to 27.8)	
Large	731	328 (89.9)	287 (79.1)	10.8 (5.3 to 16.3)	
Prior CVCs					0.547
Present	243	107 (88.4)	96 (79.3)	9.1 (-0.9 to 19.1)	
Absent	764	338 (89.4)	294 (76.8)	12.7 (7.1 to 18.2)	
Prior PICCs					0.992
Present	124	63 (91.3)	44 (81.5)	9.8 (-4.1 to 23.8)	
Absent	880	381 (88.8)	344 (76.8)	12.0 (6.9 to 17.2)	
PICCs arm side					**0.021**
Left	228	92 (81.4)	87 (77.7)	3.7 (-7.7 to 15.2)	
Right	777	352 (91.4)	302 (77.2)	14.2 (8.9 to 19.5)	
PICC body posture					0.883
Supine	868	381 (88.8)	333 (76.4)	12.4 (7.2 to 17.6)	
Semirecumbent	137	64 (92.8)	57 (85.1)	7.7 (-4.3 to 19.6)	

*Adult weight measures. The national health and family planning commission of the People's Republic of China. 2013-04-18. http://www.nhfpc.gov.cn/ Last accessed on 2015-Sep-2.

BMI: Body Mass Index, CI: Confidence Interval, CVCs: Central Venous Catheters, IC ECG: Intracavitary Electrocardiogram, PICCs: Peripherally Inserted Central Catheters.

## Discussion

To our knowledge, this EGG study is the first randomized controlled study to compare IC ECG and landmark techniques for PICC placement in adult patients with cancer. Our study demonstrates that PICCs inserted using IC ECG technique are positioned relatively better. The comparative advantage was observed in almost all prespecified patient subgroups undergoing chemotherapy. This method may provide a possibility to avoid catheter tip placement outside of the SVC.

In the last 20 years, PICCs have come to play an important role in intravenous therapy and treatment because of a few key advantages over traditionally placed CVCs [1, 26]. Currently, several methods are available for the positioning of PICC catheter tips [1, 5, 13, 27]. Of them, the most frequently used techniques are traditional anatomical landmark guidance, followed by IC ECG guidance [5, 27]. The latter technique, which uses ECG to guide CVC placement, emerged in 1949 [28], and a number of non-randomized studies have recently been published regarding its performance and safety. These studies all agree that ECG technique performs well for the positioning of PICCs [1, 13]. Unfortunately, there are very few head-to-head studies comparing these techniques. In a very recent small comparative study by Baldinelli et al. [27] (n = 90 PICCs), the authors reported significant benefits from ECG guidance versus landmark guidance, but recognized non-randomization as one of major limitations of the methodology [27]. At the time of designing the present study, this report was not yet published. Our study aimed to confirm the benefits of IC ECG technique for PICC placement.

The malpositioning of PICCs occurs rather frequently, but the exact rate can vary greatly. Trerotola et al. [29] reported in a review of medical records that 10% of tips in 1,654 PICC attempts were malpositioned. Fricke et al. [30] summarized 843 consecutively placed pediatric PICCs, of which 85.8% were associated with non-central PICC tip positioning initially. Minkovich et al. [31] reported an incidence of aberrant positioning or suboptimal positioning of 35% and 13%, respectively, using a database of 269 patients. Therefore, for robust estimation in adult patients with cancer, we tripled our study size from the initial calculation. Accordingly, advantages might be seen for IC ECG technique among larger subgroups. Our study showed that landmark guidance had a relatively low overall success rate (77.4%), which was consistent among nearly all subgroups. In a Cochrane review with 18 included studies (n = 1792) comparing ultrasound and landmark guidance via internal jugular vein cannulation for CVCs (direct puncture) [22], landmark guidance achieved an overall success rate of 88.7%, but with varying rates in individual studies. Our lower rate in the landmark guidance group might also result from the different veins cannulated [22] and different vascular statuses even in the same Chinese setting [5]. Similarly, a lower overall success rate of 89.2% occurred in the IC ECG group, which was due to a lower success rate (81.4%) in the patients with PICCs inserted from the left arms. This decrease in success on the left arm may be explained by a higher P-wave force, as this could confuse operators when trying to accurately secure the catheter tip at the desired location. When catheters were advanced into the SVC from the left side, the PICC tip tended to stay closer to the right wall of the SVC due to the stretching force introduced by the catheter. On this occasion a smaller angle of the PICC tip—resultant atrial depolarization vector developed and then a consequent higher P-wave force occurred.

Although this randomized study demonstrated an expected and consistent superiority of IC ECG technique, we must acknowledge that this study is open-labeled given the totally different techniques used. Nonetheless, in order to avoid introducing bias, the confirmative X-ray was centrally reviewed and the reviewers were blinded to the PICC technique used in each patient. Secondly, in view of the focus of the current report, we did not provide any data regarding cost, long-term efficacy, and safety. PICC-related complications will be a topic of research for us in the future. Like a similar report observed in another large multicenter study (1,444 patients) in European [32], our current analysis did not report any catheter-related adverse events or complications during the PICC manipulation in either group. Third, the use of IC ECG technique is subject to a standard P-wave identified on a surface ECG. For this reason, about 7% of cases of any disease might have to resort to other PICC placement methods [13]. The IC ECG catheter tip positioning technology usually relies on normal electrophysiology. Therefore, if those patients with structural heart diseases and similar conditions contraindicated for chemotherapy were identified and counted out, most cancer patients in need of chemotherapy would benefit from this PICC technique.

In conclusion, this randomized study demonstrated the superior efficacy in terms of first-attempt success rate of IC ECG technique for PICC placement guidance in adult patients with cancer undergoing chemotherapy. There were no significant safety concerns reported for catheterization using the two techniques. Moreover, it might be of clinical relevance that the significant beneficial difference observed almost completely disappeared in patients with PICCs inserted from the left arms.

## Supporting information

S1 File Study protocol, English version. (PDF)

S2 File Study protocol, Chinese version. (PDF)

S3 File CONSORT 2010 Checklist. CONSORT: CONsolidated Standards of Reporting Trials.
(DOC)

S1 Fig Evolving ECG monitor photographs among the same individual patient: (A): surface; (B) SVC; (C) RA; (D) SVC-RA junction. ECG: electrocardiograph; RA: right atrium; SVC: superior vena cava.
(TIF)

S1 Table Comorbidities and medical history (N = 1,007). (DOCX)

S2 Table Table PICC insertion characteristics (ITT, N = 1,007). ITT: intent-to-treat; PICC: peripherally inserted central catheter.
(DOCX)

S3 Table Catheter tip placement at first attempt by ultra-sound or chest x-ray confirmation and corresponding rates of outcome measures in subgroup patients with major prior surgery (N = 551). (DOCX)

S4 Table Catheter tip placement at first attempt by ultra-sound or chest x-ray confirmation and corresponding rates of outcome measures in subgroup patients with basilic vein used for PICCs (N = 892). PICCs: peripherally inserted central catheters.
(DOCX)
