# Supplementary material for: Superior success rate of intracavitary electrocardiogram guidance for peripherally inserted central catheter placement in patients with cancer: A randomized open-label controlled multicenter study
Source: PLoS One. 2017 Mar 9;12(3):e0171630. doi: 10.1371/journal.pone.0171630 (PMC5344315; doi:10.1371/journal.pone.0171630)
Supplement: S1 File — (PDF) [file pone.0171630.s001.pdf]

PROTOCOL SYNOPSIS

**Study rationale:**	The use of peripherally inserted central catheters (PICCs) has increased significantly for cancer patients receiving chemotherapy. For these patients, PICCs afford many advantages with regards to complications and treatment convenience.<br><br>Current practice utilizes the estimated length of the catheter from puncture site to the junction of superior vena cava / right atrium (SVC-RA) for guiding tip placement. Next the catheter tip placement was confirmed by radiographic imaging prior to use of the line for administration of chemotherapy medications. In this case, the catheter is often mal-positioned and requires adjustment and repeat radiographic imaging in order to ensure proper placement, ideally at the SVC-RA junction. These potentially additional procedures are time-consuming and also expose patients, nurses and physicians to radiations.<br><br>Intracavitary electrocardiogram with an electrode placed inside the catheter during insertion has shown identifiable changes in P-wave, which are sufficient to guide PICC tip placement. Moreover, less procedural time and radiation are expected. However, there is lack of randomized controlled studies to compare these methods.
**Title of the study:**	An Intracavitary Electrocardiographic System for Real-time Positioning Peripherally Inserted Central Catheter Tip (PROJECT_EGG)

**Study number:**	2015-001
**Coordinating investigator:**	Yuan Ling<br>The Affiliated Nanjing Drum Tower Hospital of Nanjing University Medical School
**Study centre(s):**	**Eight (8) sites as follows:**<br>The Affiliated Nanjing Drum Tower Hospital of Nanjing University Medical School<br>Jiangsu Cancer Hospital, Nanjing, Jiangsu Province, China<br>People's Hospital Affiliated to Jiangsu University, Zhenjiang, Jiangsu Province, China<br>Nanjing Hospital Affiliated to Nanjing Medical University, Nanjing, Jiangsu Province, China<br>The First Affiliated Hospital of Soochow University, Suzhou, Jiangsu Province, China<br>The Second Affiliated Hospital of Nanjing Medical University, Nanjing, Jiangsu Province, China<br>The Second Affiliated Hospital of Soochow University, Suzhou, Jiangsu Province, China<br>Nanjing Jinling Hospital, Nanjing, Jiangsu Province, China
**Clinical phase:**	Phase 4
**Objectives:**	The primary purpose of this study is to compare a new intracavitary electrocardiogram (ECG) guiding method for real-time positioning the tip of three-way valve type peripherally inserted central catheters versus conventional landmark method in terms of first-attempt success rate.

	In addition, we would like to investigate symptomatic thrombosis incidence and its risk factors after three-valve PICC implanted among patients with malignant tumours; to clarify procedure time used and cost of the new intracavitary ECG guiding method and to explore the maximal P-wave amplitude of intracavity ECG real-time positioning technology and its predictive factors.
**Study design:**	Multicenter, open-label, randomized controlled parallel study

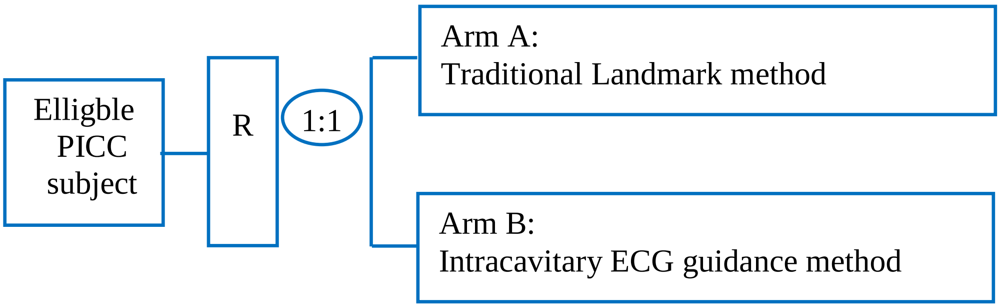

**Number of patients:**	1,000 subjects
**Diagnosis and main criteria for inclusion:**	Eligible inpatients or outpatients had to meet the following criteria:<br>• Aged 18 to 80 years;<br>• Had malignant tumours that require periodical infusion of chemotherapy drugs via three-valve PICCs;<br>• Had normal P wave according to surface ECG recordings prior to PICC insertion.<br>• Patients also had to provide signed informed consent to participate.<br>Patients with the following conditions were

	excluded from the study:<br>• Heart diseases, such as valvular heart disease, atrial fibrillation, supraventricular tachycardia, pulmonary heart disease or<br>• Having a pacemaker and post cardiac surgery which may affect P waves; or<br>• Inability to lie in the prostrate or semi-supine position.
**Study product, dose and mode of administration:**	**Intracavitary electrocardiogram (IC ECG) guidance method:**<br>The ECG-guided tip positioning method will be used to identify catheter tip location during the procedural placement of the catheter. This method involves an ECG monitor which is connected to the guidewire used for catheter placement. The changes in the ECG p-wave will guide correct PICC placement. An X-ray radiograph will be conducted to confirm the tip location after PICC placement.<br>According to our pilot study, after puncture was done, the catheter was advanced gently with the ECG P -wave shape and amplitude monitored sequentially: (1) when the catheter tip is located outside of the SVC or just entering the SVC, no obvious change in P-wave occurred relative to surface ECG; (2) when continuing to slowly advance catheter along SVC, P-wave would enlarge gradually; (3) when catheter tip reached the atrium, the following changes in P-wave would be observed: low-frequency wave would be shifted into high-frequency; shape would be

	changed from obtuse to highly sharp with either PR segment moving down or negative P-wave starting to appear (amplitude ⩾1 mm). Immediately the catheter was pull back slowly until P-wave returned to a low-frequency obtuse shape and with no negative wave, which then indicating that catheter tip is located in the lower third SVC section or at the CAJ.
**Reference product, dose and mode of administration:**	**Conventional Landmark method:**<br>The method will estimate the length of the catheter from puncture site to the junction of superior vena cava / right atrium (SVC-RA). Tip location is placed according to the estimated length but later on will be confirmed by chest X-ray performed after the placement procedure. If the tip location is not at an ideal place, additional procedures and X-ray radiograph would be required in order to ensure proper tip location.
**Criteria for evaluation:**	The confirmative x-ray data of tip location in the study were read separately by two independent radiologists. If inconsistent findings occurred, a third radiologist would further check x-ray recordings and concluded to prefer the choice of majority.
**Efficacy assessments:**	• **The first-attempt success rate** as primary efficacy endpoint was defined as proportion of patients whose catheter tips are in the SVC or at the CAJ during the first attempt of PICC insertion via confirmation by chest x-ray.<br>• **Optimal target rate:** If the catheter tips are in

	the lower third SVC section or at the CAJ, their placements are regarded as optimal.<br>• **Right atrium rate:** the proportion of patients whose catheter tips are finally in the RA space at the first attempt.<br>• **Max P-wave amplitude:** P-wave often reaches the peak when the catheter tip sits at the junction of SVC and RA.
**Safety assessments:**	Safety profile data will be collected as follows:<br>• PICC - related symptomatic thrombosis: any thrombosis events reported by subjects and with relationship as “related” or “indeterminate” at investigator’s discretion.<br>• Any other adverse events and complications;<br>• Heart rate and blood oxygen were monitored in the IC ECG group alone.<br>• In addition to demography and medical condition data, physical and laboratory examinations were performed prior to the start of and /or during the PICC operation.
**Health economics evaluation:**	Time and cost consumed for PICCs procedure
**Statistical methods:**	All randomly assigned patients were included as intention-to-treat (ITT) population into the primary analysis. No any missing data were imputed and as-observed rule was implemented.<br>The primary efficacy parameter of first-attempt

	success rate was compared using a Pearson's Chi-square test at two-sided 5% significance level. 95% confidence intervals (CIs) for success rate and rate difference were provided to indicate precision. The CIs used normal approximation or Clopper-Pearson's exact method to estimate. Interactions between planned covariates and PICCs technique were evaluated using logistic model. The P values for interaction were reported. The prespecified stratifications were based on the following covariates: gender; age group; body mass index (BMI) classification; cancer duration since diagnosis; current cancer metastasis status; prior chemotherapy; prior chest radiotherapy; smoking status; current activity amount; prior CVCs; prior PICCs; arm side of PICCs and body posture of PICCs. Additional analyses on selected subpopulations were carried out. Other statistical tests will be utilized as appropriate for other endpoints.<br><br>Assuming a first-attempt success rate 88% for anatomical landmark as reported in previous studies, a sample size of 135 patients per group have 90% power to detect an increase in rate of 10%. With an estimated 10% patients of drop out from study, 300 patients have to be randomized. Given prespecified potential subgroup analyses, the sample size was enlarged to 1,000 randomized patients (500 in each group).
**Duration of study:**	• Study Start Date: March 2015<br>• Estimated Primary Completion Date: June

	2015<br>• Estimated Study Completion Date: October 2015<br>All subjects will be followed up for four months after first PICCs or until catheter's removal from the body. Subjects will be free to withdraw from the study at any time.<br>• Final report: Q2 2016
**Protocol version and date:**	Version 1.1, 01-Febuary-2015
