# Supplementary material for: Superior success rate of intracavitary electrocardiogram guidance for peripherally inserted central catheter placement in patients with cancer: A randomized open-label controlled multicenter study
Source: PLoS One. 2017 Mar 9;12(3):e0171630. doi: 10.1371/journal.pone.0171630 (PMC5344315; doi:10.1371/journal.pone.0171630)
Supplement: S2 File — (PDF) [file pone.0171630.s002.pdf]

临床研究方案

方 案 摘 要

项目名称	一种新型腔内ECG引导系统在三向瓣膜式PICC尖端实时定位的多中心临床研究
研究目的	主要目的：<br>评价恶性肿瘤患者中三向瓣膜式 PICC 新型腔内 ECG 实时定位技术与传统的体表预测长度盲送方法相比，PICC 尖端一次性定位准确率；<br>次要目的：<br>a. 了解恶性肿瘤患者置入三向瓣膜式 PICC 后症状性血栓发生率及其危险因素；<br>b. 比较恶性肿瘤患者中三向瓣膜式 PICC 新型腔内 ECG 实时定位技术与传统的体表预测长度盲送方法相比的耗时和经济性<br>c. 了解恶性肿瘤患者采用腔内 ECG 实时定位技术时 P 波的最大幅度及其影响因素<br>d. 了解恶性肿瘤患者采用传统的体表预测长度盲送方法定位时一次性定位准确的影响因素<br>e. 通过对比 ECG 前后变化来预测 PICC 尖端维持定位准确
研究设计	前瞻性随机对照开放的多中心研究
病例总数	1000例，8个研究中心
病例选择	*入选标准：*<br>1. 恶性肿瘤拟行周期性输注化疗药物需置入三向瓣膜式 PICC 患者<br>2. 年龄>18 岁至＜80 岁的患者<br>3. 置管前基础 ECG 记录显示正常 P 波<br>4. 同意参与本研究，并签署 PICC 置管和本研究知情同意书
	排除标准<br>5. 心脏疾患，如瓣膜性心脏病、心房纤颤、室上性心动过速、肺源性心脏病或有植入心脏起搏器及心脏外科术后等可能影响 P 波改变的患

	者。<br>6. 不能平卧或半卧位的患者
治疗方案	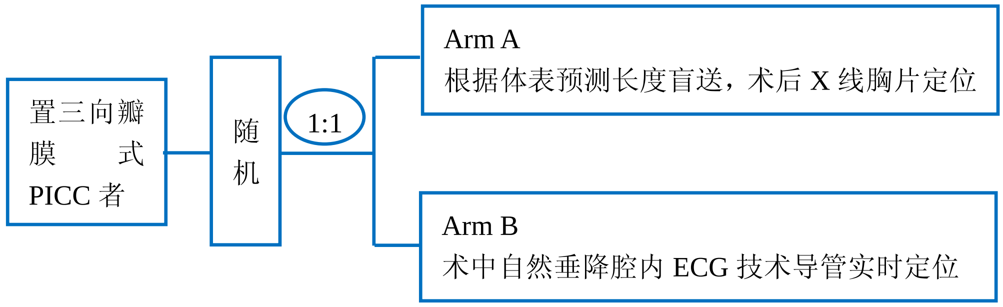
疗效评定	*有效性评价指标（主要疗效指标和次要疗效指标）*<br>主要疗效指标：<br>一次性定位准确率：初次置管后通过 X 线胸片确定导管尖端位于 SVC/RA 连接的上下 2cm 范围内。此为 PICC 尖端目标的安全区域。<br>次要评价指标：<br>可行性：能够通过盐水柱法在 PICC 管腔内建立的盐水电极，引导出患者腔内心电图 ECG 的几率。<br>ECG 中 P 波的最大幅度：当导管尖端到达 SVC/RA 交界处（解剖位置）时，P 波往往达到最高峰。
	安全性评价指标<br>PICC 相关的症状性血栓：由患者报告的血栓事件，研究者判断其因果关系为与 PICC 有关或者不明确。<br>其它不良事件：患者应用腔内 ECG 技术过程中有无不适主诉、心电监护有无心律失常表现。
卫生经济学评价	耗时和经济性：使用此新型腔内 ECG 引导系统或传统方法的完成定位所需要的操作时间与费用成本。
统计方法	考虑到主要研究目和重要次要研究目的，本研究最终样本量总共需要 1,000 受试者。本研究将采用全分析人群作为主要疗效分析人群，其包括所有随机化后置入导管的受试者。

	本研究将提供描述性统计量，连续性变量时包括均数、标准差、中位数、四分位间距、最小和最大值；分类变量时提供发生频数和百分比，以及必要的 95%的可信区间。<br>本研究主要终点将采用卡方分析进行统计检验。如果统计检验获得双侧 P 小于 0.05，则该无效假设将被拒绝，将接受备择假设，同时计算两组差率的 95%可信区间，以确定 ECG 实时指导定位技术在方面的优效性。还将进行多重亚组分析，看不同亚组中 ECG 实时指导定位技术的优效情况。还将采用 Logistic 回归对其独立的影响因素进行探讨。<br>症状性血栓发生率将整体统计描述其发生情况，并探讨不同亚组人群中发生率的变化。并将采用 Logistic 回归对其独立危险因素进行探讨。同时采用 Kaplan-Meier 曲线对血栓发生时间进行统计描述等。
研究期限	首次置管后随访观察4个月

一、研究目的

主要目的：

评价恶性肿瘤患者中三向瓣膜式 PICC 新型腔内 ECG 实时定位技术与传统的体表预测长度盲送方法相比，PICC 尖端一次性定位准确率。

次要目的：

a. 了解恶性肿瘤患者置入三向瓣膜式 PICC 后症状性血栓发生率及其危险因素；

b. 比较恶性肿瘤患者中三向瓣膜式 PICC 新型腔内 ECG 实时定位技术与传统的体表预测长度盲送方法相比的耗时和经济性

c. 了解恶性肿瘤患者采用腔内 ECG 实时定位技术时 P 波的最大幅度及其影响因素

d. 了解恶性肿瘤患者采用传统的体表预测长度盲送方法定位时一次性定位准确的影响因素

e. 通过对比 ECG 前后变化来预测 PICC 尖端维持定位准确

二、研究设计类型、原则与实验步骤

1. 研究设计

本研究为一项为期 4 个月的前瞻性随机对照开放的多中心临床研究。适应症为：接受超声引导结合改良赛丁格法行前端闭合式 PICC 置管患者。

根据 2014 年我肿瘤中心数据显示及相关数据常规 PICC 置管时一次性定位准确率为 88%，而腔内 ECG 的 PICC 尖端实时定位技术导管易位率文献报道极低，因此根据这些参数假设以及潜在的受试者脱落情况，两组需要样本量合计 300 例受试者。考虑到亚组人群分析以及本研究次要目的还计划了解恶性肿瘤患者置入三向瓣膜式 PICC 后症状性血栓发生率，因此，本研究扩大样本量，进行一项多中心（8 个中心）参与的大样本临床应用研究，预计入组患者总计 1,000 例。

本研究除置入导管基线访视外，还将持续收集并发症信息 4 个月，并在第 4 个月时进行一次腔内 ECG 检查，并进行 X 线检查确认 PICC 尖端位置。基线访视收集的信息主要包括人口学特征，身高、体重、既往疾病、合并疾病和用药史，肿瘤疾病情况，中心静脉置管史，PICC 置管史，置管前血液学检查指标，PICC 置入记录，腔内 ECG 引

导下 PICC 置入时额外收集的信息，拔管信息等。

2. 研究步骤

患者按 1:1 比例按研究中心分层随机分配进入试验组或对照组，分别接受常规置管定位技术（根据体表测量决定导管留置长度+置管后 X 线胸片检查+必要时根据 X 线胸片检查结果调整导管留置长度）或术中自然垂降法腔内 ECG 的 PICC 尖端实时定位技术。

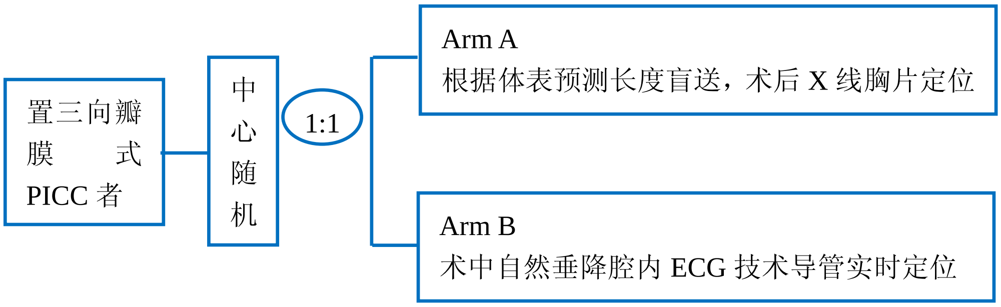

三、病例选择和分配

接受超声引导结合改良赛丁格法行前端闭合式 PICC 置管患者

1. 入选标准

a. 恶性肿瘤拟行周期性输注化疗药物需置入三向瓣膜式 PICC 患者

b. 年龄>18 岁至＜80 岁的患者

c. 置管前基础 ECG 记录显示正常 P 波。

d. 同意参与本研究，并签署 PICC 置管和本研究知情同意书。

2. 排除标准

a. 心脏疾患，如瓣膜性心脏病、心房纤颤、室上性心动过速、肺源性心脏病或有植入心脏起搏器及心脏外科术后等可能影响 P 波改变的患者。

b. 不能平卧或半卧位的患者。

3. 治疗分配方法

本研究将采用按研究中心分层的排列区组随机方法。为了尽量避免该开放试验中分组的被预见性，随机的区组大小预设为可变化。两组患者的分配比例 1:1。按研究中心分层的随机表和清单，将统一提供给各个研究中心的研究协调员。研究协调员将

按照患者满足研究入组标准的时间顺序遵照随机清单依次分配受试者。直到分配前，受试者和其它研究人员不允许知晓受试者随机分配的序列。

将由第三方专业机构产生随机表并制作随机信封。

四、研究方法与技术路线

1. 操作者培训及考核

此研究由各研究中心中经过培训及考核的 2 名肿瘤及静疗专科护士完成。培训方法为：由心脏介入科主任医师给所有操作护士进行“ECG 定位 PICC 尖端操作能力”的培训，详细讲解体表和腔内 ECG 的基础知识，ECG 导管尖端定位技术的适应症、ECG 设备的连接与使用、导管尖端位置与 P 波的关系与判断。培训后由该主任医师对所有操作护士进行 ECG 定位 PICC 尖端操作能力考核，确认所有操作护士已经具备 ECG 定位 PICC 的能力。

2. 人员分工

置管操作者：每个中心由同一名肿瘤专科护士负责，包括置管长度体表测量、PICC 穿刺置管、腔内心电图引导系统的连接、心电监护波形观察、导管位置的调整和确定、导管固定。

记录员：每个中心由同一名静疗专科护士负责，包括打印记录置管前原始心电图、导管尖端进入心房的心电图（出现双向 P 波或者 P 波振幅回落）、导管尖端从心房退出至 SVC/RA 交界处的心电图（P 波振幅高峰位置的心电图）。记录操作时间、盐水用量、X 线胸片检查次数以及其他各项信息资料的记录、统计资料录入和双人核对。

协调员：由同一名副主任医生负责，包括随机分组，保证患者、操作者在置管前都不知道患者入对照组还是观察组、督促量表填写，每位患者的相关研究资料进行编码将资料分别放入资料袋中由协调员保管，协调员不参与方案的设计与实施。

并发症评价者：每个中心为同一名经过导管维护及并发症识别及处理的临床总责护士。评价者均不参与方案设计与实施。

X 线胸片评读者：为同一名放射科主管技师。评读均不参与方案设计与实施。

统计者：由同一名具有医学统计学培训的医生负责，统计资料由两人并核对。

3. 物品

所有仪器及用物统一。科曼 C58 系列床旁心电监护仪（采用 3 电极心电监护模式），

无菌心电导联线，Bard PICC（ Groshong 4Fr）1 套，改良赛丁格穿刺包、PICC 置管包各 1 套，柯惠电极 3 个，20ml 注射器 2 支，无菌手套、0.9%NaCl 注射液（100ml）、一次性使用输液器、10.0cm×11.5cm 的 3M 敷料及皮尺各 1 个。

4. 环境

所有置管操作均在各个肿瘤中心置管室进行。室内所有人员关闭手机。超声下 PICC 穿刺成功进入血管后即关闭超声仪避免对心电监护仪的电磁干扰。

5. 具体干预措施

a. 对照组（常规超声引导结合改良塞丁格技术 PICC 置管法）:

按照超声引导结合改良塞丁格技术操作规范在上臂中1/3段行PICC置管。患者取仰卧位，拟穿刺上肢外展90°，在上臂中1/3段行PICC置管。导管预留长度根据置管常规进行体表测量决定，方法为自穿刺点测量至右胸锁关节再向下反折至第三肋间隙。置管后均行X线胸片检查定位，根据X线胸片定位结果必要时进行导管位置调整，导管位置调整后需再次行X线胸片检查定位，直至导管尖端到达安全位置为止。

b. 试验组（常规置管加自然垂降盐水柱法引导腔 ECG 行 PICC 尖端定位技术）:

具体操作步骤如下。①连接床旁三导联心电监护仪， 3个心电电极片分别贴于左侧锁骨下（LA）、右侧锁骨下（RA）、和左侧锁骨中线肋骨下缘（LL）体表皮肤，将心电监护仪调至Ⅱ导联，记录体表心电，确保患者心电图具有P波；②按照超声引导结合改良塞丁格技术操作规范在上臂中1/3段行PICC置管；③PICC穿刺成功后导管送入体内20cm时，将事先悬挂于床旁输液架上（高度同密闭式输液要求，液平面距穿刺部位60-100cm高度）连接好生理盐水软袋的无菌输液器乳头与PICC内置可支撑冲洗导丝尾端的厄尔接头连接（见图7）；④将另配的单包装无菌鳄鱼嘴夹导联线稳妥夹持于PICC内置导丝尾端的金属暴露部位（见图7）；⑤取下心电监护仪上原先与患者相连的右锁骨下（RA）导联线，替换为已与PICC连接的无菌导联线；⑥护士缓慢打开输液器调节器（经预试验，滴数为20滴/分），生理盐水注入PICC,按照产品说明书要求，此时PICC管内压力大于80mmHg，导管末端三向瓣膜向外开放形成与人体静脉血液相连的生理盐水柱，将盐水柱作为患者腔内盐水电极，引导出心电图变化显示于监护仪显示屏上；⑦护士一边缓慢轻柔送管，一边密切观注监护仪显示屏，通过判断P波振幅变化辅助导管定位。

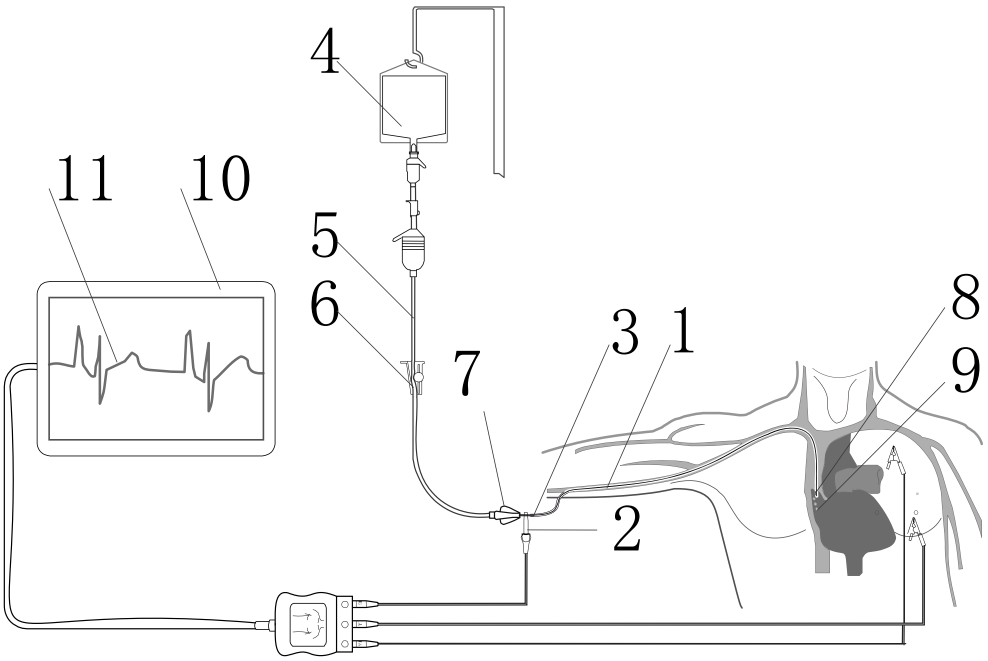

①Groshong PICC；②RA 导联连接头；③内置导丝尾端金属暴露部分；④生理盐水瓶；⑤输液器；⑥调节器；⑦可支撑冲洗导丝尾端的厄尔接头；⑧盐水流柱；⑨三向瓣膜；⑩心电监护显示器；⑪心电图波型

图 3　**自然垂降法盐水柱腔内 ECG 技术辅助 Groshong PICC 尖端定位连接示意图（申请人绘制）**

（专利号 ZL 2014 2 0436176.7）

整个方案实施过程中，对照组和试验组的其他护理，如：置管前和置管后的健康教育，出院指导，导管维护，并发症预防、观察及处理等均按原有护理常规处理。

五、疗效评定标准

1、可行性：能够通过盐水柱法在 PICC 管腔内建立的盐水电极，引导出患者腔内心电图 ECG 的几率。

2、精确性：对比置管中运用 ECG 技术行导管尖端定位与在置管后运用 X 线胸片技术行导管尖端定位的匹配度。

①导管易位：导管尖端不在上腔静脉的下1/3和心房的上1/3区间。

②导管位置正确：导管未易位，但是不在ECG技术引导预计到达的位置

③导管位置匹配：导管在ECG技术引导下到达预计位置（本研究预计到达位置为2011版INS指南中规定的上腔静脉下1/3段靠近右心房连接处）。

④放射学判断标准：SVC的放射学标记为右侧支气管角至心影右侧缘的距离段[28]。SVC/RA交界处的放射学标记为气管隆突下3cm处；SVC下1/3段的放射学标记为气管隆突下至第一个3cm处的距离段；RA上1/3段的放射学标记为气管隆突下3cm-5cm的距离段[10]。一些研究者提出SVC/RA连接处是位于SVC/RA连接的上下2cm范围内，为导管留置最安全区域[5、29]。

3、　经济性：计算每例患者置管期间使用ECG技术的费用、置管后进行X线胸片的费用、置管及导管留置期间处理并发症的费用。

4、　ECG波形显示连续性：记录每例患者ECG引导过程中波形中断次数。

5、　生理盐水用量：以ml为单位，取整数。从置管成功导管送入20cm起连接生理盐水开始计算到根据P波变化最终确定导管留置位置为止,计算腔内ECG引导过程中生理盐水用量。

6、　流程便捷性：评价操作护士对每例患者腔内ECG引导方法操作流程便捷性的满意度。采用Likert 5级评分制（非常不满意=1，比较不满意=2，不确定=3，比较满意=4，非常满意=5。

7、　并发症发生率：从置管到导管拔除期间所有并发症发生比例。初次置管后观察 4 个月。

七、数据安全监查

所有不良事件均详细记录，恰当处理并追踪直到妥善解决或病情稳定，按照规定及时向伦理委员会、主管部门、申办者和药品监督管理部门报告严重不良事件与非预期事件等；主要研究者定期对所有不良事件进行累积性回顾，必要时召开研究者会议评估研究的风险与受益；大于最小风险的研究将安排独立的数据监查员对研究数据进行监查，高风险研究将建立独立的数据安全监察委员会对累积的安全性数据以及有效性数据进行监查，以做出研究是否继续进行的建议。

八、统计学处理

1. 统计学考虑

将由主要研究者或者其指定代表开展统计分析工作。根据实际需要，将在数据清理完成前确定研究的统计分析计划。

2. 样本量

为了获得足够的疗效和安全性数据，根据研究目的确定此项研究的最终样本量。

根据主要研究目的，本研究中恶性肿瘤患者采用三向瓣膜式 PICC 新型腔内 ECG 实时定位技术，与传统的体表预测长度盲送方法相比，PICC 导管尖端一次性定位准确率预计将从 88%提高到 98%以上。因此，假设无效假设率差为 0%，采用双侧显著性水平 α=0.05 进行 Pearson 卡方检验，样本量每组 135 例受试者(1:1 随机分配)将有 90%的统计把握度检测出 10%（98% vs. 88%）及以上的准确率率差。考虑到约 10%的患者脱落，本研究总共需要随机 300 例受试者。鉴于事先指定的潜在亚组分析，有必要对此样本量进行适度扩大。

本研究次要目的还计划了解恶性肿瘤患者置入三向瓣膜式 PICC 后症状性血栓发生率。根据文献，不同研究人群中 PICC 相关血栓的报告发生率变化非常大，PICC 相关的症状性血栓发生率介于 1%－25.7%。因此，在一定的误差下了解不同的发生率，需要不同的研究样本量(表 1)。若假设本研究期间恶性肿瘤患者置入三向瓣膜式 PICC 后症状性血栓发生率约 10%，样本量 865 例受试者将有 95%的把握预计的症状性血栓发生率（10%）的相对误差小于 20%(绝对误差为±2%)，另考虑到约 12-14%的患者脱落，本研究总共需要入组 1,000 例受试者。

综上两个研究目的考虑，本研究最终样本量总共需要 1,000 受试者。

表 1 误差变化时不同血栓发生率所需要的样本量（把握度 95%）

发生率	相对误差范围				
	±10%	±15%	±20%	±25%	±30%
5%	7,300	3,245	1,826	1,169	812
10%	3,458	1,538	865	554	385
15%	2,178	968	545	349	243
20%	1,538	684	385	247	172
25%	1,153	513	289	185	129
30%	897	399	225	144	101

3. 统计分析人群

本研究将采用全分析人群作为主要疗效分析人群，其包括所有随机化后置入导管的受试者。分析置入三向瓣膜式 PICC 后症状性血栓发生率及其危险因素，比较耗时和成本时，同样采用全分析人群。受试者将按照其随机分配的置管方法进行分组分析。

另外对进行腔内 ECG 实时定位技术组受试者，进行其 P 波的最大幅度及其影响因素以及对比受试者 ECG 前后变化来预测 PICC 尖端维持定位准确的分析。

还对采用传统的体表预测长度盲送方法定位的受试者，进行其一次性定位准确的影响因素的分析。

4. 统计分析变量与统计方法

本研究将提供描述性统计量，连续性变量时包括均数、标准差、中位数、四分位间距、最小和最大值；分类变量时提供发生频数和百分比，以及必要的 95%的可信区间。必要时，在统计分析前会对原始数据进行转换。

主要终点

本研究的主要终点为初次置管后，导管到达预计位置的比例（本研究预计到达位置为 2011 版 INS 指南中规定的上腔静脉下 1/3 段靠近右心房连接处)，即一次性定位准确率。

本研究主要终点将采用卡方分析进行统计检验。将对如下假设进行统计检验以证实 ECG 实时指导定位的优势：

H_0: $P_1-P_0=0$ vs. H_1: $P_1-P_0\neq 0$,

其中，P_1 表示 ECG 实时指导定位组的一次性定位准确率，P_0 表示对照组的一次性定位准确率。如果统计检验获得双侧 P 小于 0.05，则该无效假设 H_0 将被拒绝，将接受备择假设 H_1，同时计算两组差率的 95%可信区间，以确定 ECG 实时指导定位技术在方面的优效性。

还将进行多重亚组分析，看不同亚组中 ECG 实时指导定位技术的优效情况。亚组分析的变量包括年龄分组、性别、体重指数、新老肿瘤患者、合并疾病、疾病治疗史、化疗史、吸烟情况、活动情况、PICC 置管史、中心静脉置管史、研究中心等。统计分析计划中将提供必要的具体分组定义。还将采用 Logistic 回归对其独立的影响因素进行探讨。

次要终点

症状性血栓发生率：将整体统计描述其发生率情况，并探讨不同亚组人群中发生率的变化，合适时提供正态近似的 95%可信区间。并将采用 Logistic 回归对其独立危险因素进行探讨。同时采用 Kaplan-Meier 曲线对血栓发生时间进行统计描述等。

操作耗时和成本：以提供描述性统计量为主，必要时进行统计检验。连续性变量采用成组 t 检验或者对应的非参数方法进行检验。

P 波的最大幅度：以提供描述性统计量为主，必要时进行统计检验。连续性变量采用成组 t 检验或者方差分析进行统计分析，如果数据不呈正态分布，则采用对应的非参数方法进行检验。还将采用一般线性回归对其独立的影响因素进行探讨。适合的话，将 P 波的最大幅度与 PICC 尖端与 SVC/RA 交界的距离，采用 Pearson 线性相关或 Spearman 等级相关进行统计分析。

PICC 尖端维持定位准确：以提供描述性统计量为主，必要时进行统计检验。还将进行多重亚组分析，看不同亚组中维持定位准确的情况。并将采用 Logistic 回归对其独立危险因素进行探讨。

九、临床研究的伦理学

临床研究将遵循世界医学大会《赫尔辛基宣言》等相关规定。在研究开始之前，由伦理委员会批准该试验方案后才实施临床研究。每一位受试者入选本研究前，研究者有责任向受试者或其代理人完整、全面地介绍本研究的目的、程序和可能的风险，并签署书面知情同意书，应让受试者知道他们有权随时退出本研究，知情同意中应作为临床研究文件保留备查。研究过程中将保护受试者的个人隐私与数据机密性。

十、研究进度

2015 年 04 月-2015 年 07 月：筛选入组

2015 年 08 月-2016 年 12 月：治疗及随访

2016 年 12 月-2017 年 05 月：数据整理统计

十一、参加人员(研究中心和主要研究者列表)

中心编号	医院名称	主要研究者
01	南京大学医学院附属鼓楼医院	袁　玲
02	江苏省肿瘤医院	陈传英
03	南京医科大学第二附属医院	陈　萍
04	南京医科大学附属南京医院	吴贤翠
05	南京军区南京总医院	祁　静
06	江苏大学附属人民医院	冯玉玲
07	苏州大学附属第一医院	杨益群
08	苏州大学附属第二医院	邱珍珠

【附件 1】自然垂降法盐水柱腔内 ECG 定位技术 SOP

自然垂降法盐水柱腔内 ECG 定位技术 SOP

一、　人员准备

1、**置管操作者：**每个中心由同一名专科护士负责，包括置管长度体表测量、PICC穿刺置管、腔内心电图引导系统的连接、心电监护波形观察、导管位置的调整和确定、导管固定。

2、**记录员：**每个中心由同一名专科护士负责，包括打印记录置管前原始心电图、出现双向P波或者P波振幅回落的心电图、P波正向振幅高峰位置的心电图、记录操作时间、X线胸片检查次数以及其他各项信息资料的记录、统计资料录入和双人核对。

二、　物品准备

科曼 C58 系列床旁心电监护仪（采用 3 电极心电监护模式）1 台，无菌心电导联线 1 根，Bard PICC（ Groshong 4Fr）1 套，改良赛丁格穿刺包 1 套、PICC 置管包 1 套，柯惠心电电极 3 个，无菌手套 2 副、0.9%NaCl 注射液（100ml）1 袋、输液器 1 副 、20ml 注射器 2 副、10.0cm×11.5cm3M 敷料及皮尺各 1 个。

三、　环境准备

所有置管操作均在各个肿瘤中心置管室进行。室内所有人员关闭手机。超声下PICC穿刺成功进入血管后即关闭超声仪避免对心电监护仪的电磁干扰。

四、　操作流程

(一)　置管前

1. 连接床旁三导联心电监护仪。3 个心电电极片分别贴于左侧锁骨下（LA）、右侧锁骨下（RA）、和左侧锁骨中线肋骨下缘（LL）体表皮肤。
2. 将心电监护仪调至Ⅱ导联，确保患者心电图具有 P 波。
3. 记录体表心电图（图纸一）。

(二)　置管中

1. 按照超声引导结合改良塞丁格技术操作规范在上臂中 1/3 段行 PICC 置管。
2. PICC 穿刺成功后应用自然垂降法盐水柱腔内 ECG 技术进行导管头端定位（见图 1 自然垂降盐水柱法引导腔内 ECG 连接示意图），具体操作步骤如下。

3. PICC（1）送入体内 20cm 时，将事先悬挂于床旁输液架上（高度同密闭式输液要求，液平面距穿刺部位 60-100cm 高度）连接好连接上生理盐水软袋（4）的输液器（5）乳头与 PICC 内置可支撑冲洗导丝尾端的厄尔接头（7）连接。
4. 将另配的无菌导联线金属夹（2）稳妥夹持于 PICC 内置导丝尾端的金属暴露部位（3）。
5. 取下心电监护仪上原先与患者相连的右锁骨下（RA）导联线，替换为已与 PICC 连接的无菌导联线（2）。
6. 护士缓慢打开输液器调节器（6）（经预试验，滴数为 20 滴/分左右），生理盐水注入 PICC, 按照产品说明书要求，此时 PICC 管内压力大于 80mmHg，导管末端三向瓣膜（9）向外开放 ，形成与人体静脉血液相连的生理盐水柱（8），将盐水柱作为患者腔内盐水电极，引导出心电图变化（11）显示于监护仪显示屏（10）上。
7. 穿刺成功后，缓慢置入 PICC 导管，同时密切关注监护仪 P 波形态及振幅：当导管尖端位于上腔静脉以外静脉或刚入上腔静脉时，P 波较体表振幅及形态无显著变化；当继续沿上腔静脉缓慢置管，可观察到 P 波振幅逐渐增大，当观察到 P 波形态出现下述变化时：P 波由低频波变为高频波、形态由圆钝变得高尖且 PR 段明显下移或 P 波初始出现负向波（振幅≥1mm），说明导管头端进入心房，此时缓慢回拉导管至 P 波再次由高频波变为低频波，形态由高尖变为圆钝且无负向 P 波处,可提示导管头端位于交界区或上腔静脉下 1/3 处。以此原理指导 PICC 导管定位。特殊情况：对于置管过程中出现 M 型波形的患者，建议继续送管至 M 波型消失，观察此时 P 波形态，如若为上述形态高尖的高频波，可判断导管头端进入心房，可稍回拉，至最早恢复为 M 型 P 波或低频圆钝型 P 波为止。
8. 当显示 P 波振幅变为达到最高峰后回落或出现“双向”,打印图纸（图纸二）。
9. 缓慢回抽导管并观察 P 波形态及振幅变化。
10. 当显示 P 波再次由高频波变为低频波，形态由高尖变为圆钝且无负向 P 波处打印图纸（图纸三）。
11. 固定导管，记录长度。

（三） 置管后

1. X 线胸片进行二次确认。
2. 记录护理记录和 CRF 表

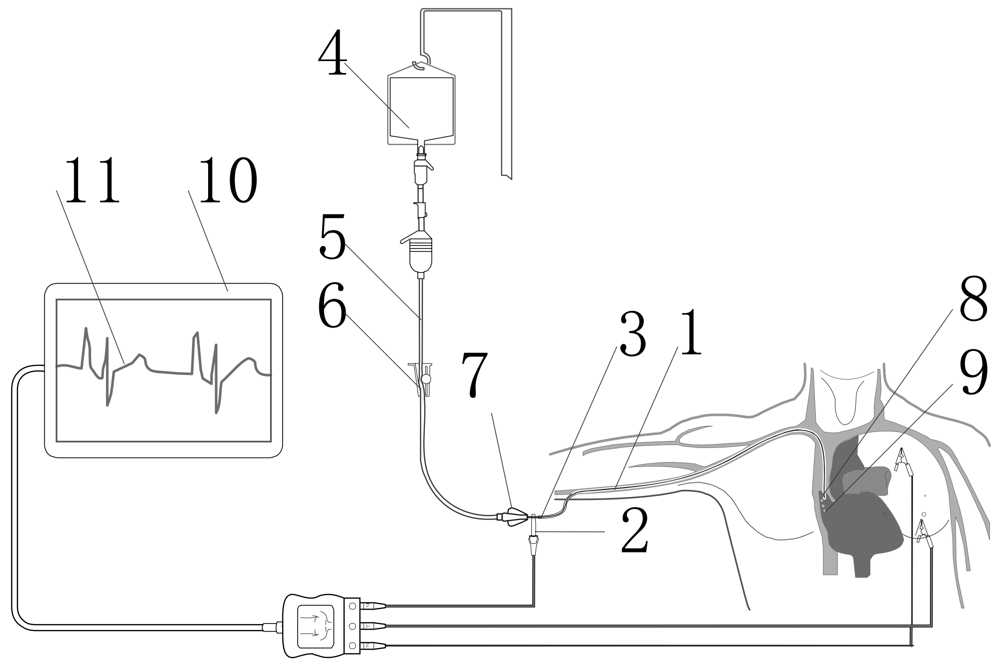

①Groshong PICC；②RA 导联连接头；③内置导丝尾端金属暴露部分；④生理盐水瓶；⑤输液器；⑥调节器；⑦可支撑冲洗导丝尾端的厄尔接头；⑧盐水流柱；⑨三向瓣膜；⑩心电监护显示器；⑪心电图波型

图1 自然垂降盐水柱法引导腔内ECG连接示意图

【附件 2】PICC 留置期间常见并发症诊断标准

PICC 留置期间常见并发症诊断标准

1. **血栓性堵管：**指导管内部或周围形成的血栓所致。临床表现为滴数减慢或滴注停止；无法冲管或抽回血。
2. **静脉血栓：**置管部位的肿胀/渗液、皮肤颜色和温度改变、麻/刺痛、形成侧支循环、不能抽血或冲管、缓慢的流速、液体自穿刺点回漏、血管超声或造影示静脉血管血栓。尤其要重视静脉血栓的隐匿症状，如病人主观感觉置管侧肢体、腋窝、肩臂部酸胀疼痛时，应给予高度重视。
3. **导管异位：**分为原发性和继发性导管定位不良及导管移动引起的导管头端异位。

   ①原发性导管异位：发生在插管过程中，导管进入各种异常位置，包括进入对侧无名静脉及锁骨下静脉，同侧或对侧颈内静脉、奇静脉、左侧或右侧胸廓内静脉、心包横隔静脉及右心房或右心室。可以通过血管超声探查、推注生理盐水、腔内心电图导引及术后 X 线定位判断导管异位情况。

   ②继发性导管异位：可能发生在留置导管期间的任意时间。胸腔内压的改变(如咳嗽、呕吐)、发生充血性心力衰竭、颈部或手臂移动、正压通气、高压注射、或冲洗导管，均有可能发生导管异位。继发性导管异位最常见部位包括颈内静脉、无名静脉、锁骨下静脉、腋窝静脉、奇静脉及右心房。临床表现为血液回抽障碍、难以或无法冲洗导管、肩部异常、胸部或背部疼痛、水肿、同侧闻及气过水声或水流声、感觉异常及液体逆流入颅内静脉窦所致的神经系统反应。

   PICC 原发与继发性导管异位均可能产生房性与室性心动过速。导管异位进入心脏的发生与手臂内收及屈曲有关。
4. **心律失常：**PICC 插入过深，以致导管尖端进入心房或心室，可诱发心率失常。临床表现为病人突然出现心慌、胸闷；心电监护显示心律失常，多为频发的室性期前收缩，后撤导管随即消失。

参考文献

[1] 王建荣，蔡虻，呼滨.输液治疗护理实践指南与实施细则[M].人民军医出版社，2010 版.
[2] 2011 版美国 INS《输液护理操作指南》.
[3] 乔爱珍,苏讯.外周中心静脉导管技术与管理[M]. 北京：人民军医出版社，2010.9.
