# Supplementary figures and images for: Superior success rate of intracavitary electrocardiogram guidance for peripherally inserted central catheter placement in patients with cancer: A randomized open-label controlled multicenter study
Source: PLoS One. 2017 Mar 9;12(3):e0171630. doi: 10.1371/journal.pone.0171630 (PMC5344315; doi:10.1371/journal.pone.0171630)

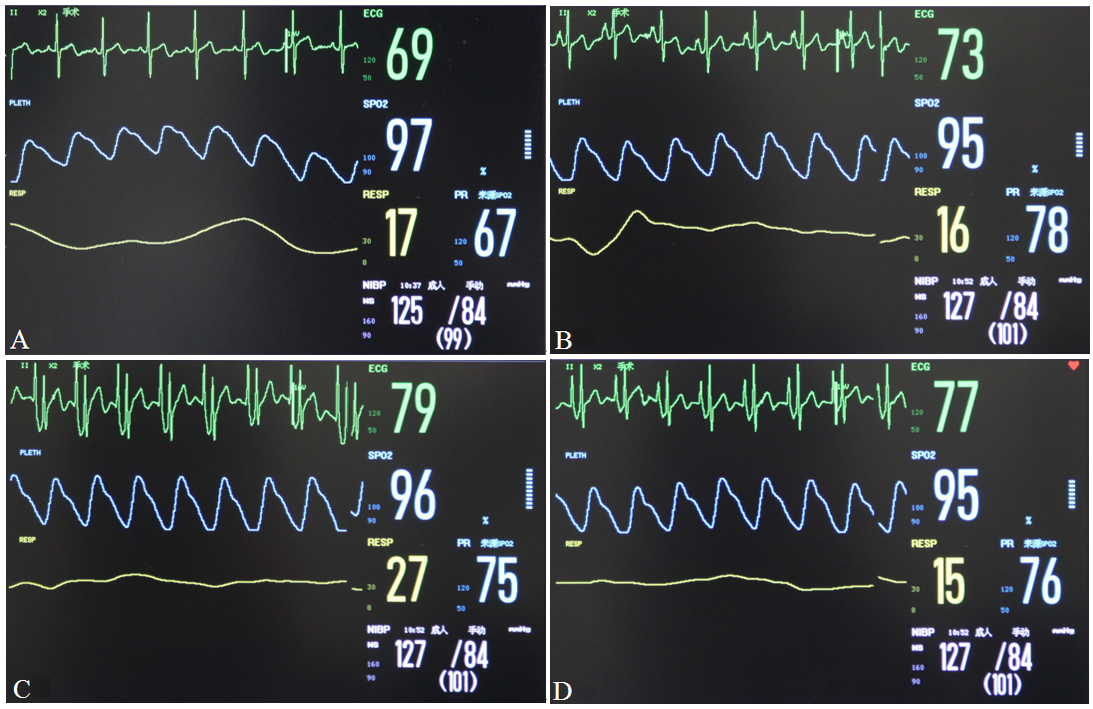

Supplement: S1 Fig — Evolving ECG monitor photographs among the same individual patient: (A): surface; (B) SVC; (C) RA; (D) SVC-RA junction. ECG: electrocardiograph; RA: right atrium; SVC: superior vena cava. (TIF) [file pone.0171630.s004.tif]
