# Supplementary material for: Superior success rate of intracavitary electrocardiogram guidance for peripherally inserted central catheter placement in patients with cancer: A randomized open-label controlled multicenter study
Source: PLoS One. 2017 Mar 9;12(3):e0171630. doi: 10.1371/journal.pone.0171630 (PMC5344315; doi:10.1371/journal.pone.0171630)
Supplement: S1 Table — (DOCX) [file pone.0171630.s005.docx]

**S1 Table: Comorbidities and medical history (N = 1,007)**

|  | **PICC method** | | **All**  **(N=1,007)** |
| --- | --- | --- | --- |
|  | **IC ECG**  **(N=500)** | **Landmark**  **(N=507)** |  |
| Patients with any comorbidities (n (%)) | 152 (30.4%) | 160 (31.6%) | 312 (31.0%) |
| Diabetes | 40 (8.0%) | 48 (9.5%) | 88 (8.7%) |
| Hypertension | 104 (20.8%) | 104 (20.5%) | 208 (20.7%) |
| Coronary heart disease | 10 (2.0%) | 15 (3.0%) | 25 (2.5%) |
| Renal insufficiency | 0 | 3 (0.6%) | 3 (0.3%) |
| COPD | 1 (0.2%) | 3 (0.6%) | 4 (0.4%) |
| Neutrophilic granulocytopenia | 7 (1.4%) | 7 (1.4%) | 14 (1.4%) |
| Other | 29 (5.8%) | 25 (4.9%) | 54 (5.4%) |
|  |  |  |  |
| Patients with any medical history (n (%)) | 283 (56.6%) | 280 (55.2%) | 563 (55.9%) |
| Thrombosis | 1 (0.2%) | 6 (1.2%) | 7 (0.7%) |
| Major bleeding | 4 (0.8%) | 2 (0.4%) | 6 (0.6%) |
| Other blood coagulation disorders | 3 (0.6%) | 1 (0.2%) | 4 (0.4%) |
| Major surgery (>1 hour) | 277 (55.4%) | 274 (54.0%) | 551 (54.7%) |
