# Supplementary material for: Superior success rate of intracavitary electrocardiogram guidance for peripherally inserted central catheter placement in patients with cancer: A randomized open-label controlled multicenter study
Source: PLoS One. 2017 Mar 9;12(3):e0171630. doi: 10.1371/journal.pone.0171630 (PMC5344315; doi:10.1371/journal.pone.0171630)
Supplement: S2 Table — ITT: intent-to-treat; PICC: peripherally inserted central catheter. (DOCX) [file pone.0171630.s006.docx]

**S2 Table: PICC insertion characteristics (ITT, n = 1,007)**

|  | **PICC method** | |  |
| --- | --- | --- | --- |
|  | **IC ECG**  **(n = 500)** | **Landmark**  **(n = 507)** | **All**  **(n = 1,007)** |
| PICC side of arm (n [%]) |  |  |  |
| Left arm | 114 (22.8%) | 114 (22.5%) | 228 (22.7%) |
| Right arm | 385 (77.2%) | 392 (77.5%) | 777 (77.3%) |
| PICC body position (n [%]) |  |  |  |
| Supine | 430 (86.2%) | 438 (86.6%) | 868 (86.4%) |
| Semirecumbent | 69 (13.8%) | 68 (13.4%) | 137 (13.6%) |
| PICC vein (n [%]) |  |  |  |
| Basilic | 438 (87.8%) | 454 (89.7%) | 892 (88.8%) |
| Cephalic | 2 (0.4%) | 4 (0.8%) | 6 (0.6%) |
| Median cubital | 6 (1.2%) | 5 (1.0%) | 11 (1.1%) |
| Other | 53 (10.6%) | 43 (8.5%) | 96 (9.6%) |
| Distance from puncture point to 3^rd^ rib (cm) |  |  |  |
| Mean (SD) | 41.9 (3.9) | 41.8 (3.8) | 41.9 (3.8) |
| PICC length (cm) |  |  |  |
| Mean (SD) | 41.2 (4.2) | 41.6 (3.9) | 41.4 (4.1) |

IC ECG: Intracavitary Electrocardiograph, ITT: Intention-To-Treat, PICC: Peripherally Inserted Central Catheter, SD: Standard Deviation.
