# Supplementary material for: Superior success rate of intracavitary electrocardiogram guidance for peripherally inserted central catheter placement in patients with cancer: A randomized open-label controlled multicenter study
Source: PLoS One. 2017 Mar 9;12(3):e0171630. doi: 10.1371/journal.pone.0171630 (PMC5344315; doi:10.1371/journal.pone.0171630)
Supplement: S3 Table — (DOCX) [file pone.0171630.s007.docx]

**S3 Table: Catheter tip placement via first attempt by ultra-sound or chest x-ray confirmation and corresponding endpoint rates with IC ECG versus landmark technique in patients with major surgery (N=551)**

|  |  | **PICC Method** | |  |  |
| --- | --- | --- | --- | --- | --- |
|  | **IC ECG**  **(N=277)** | | **Landmark**  **(N=274)** | | **p-value** |
| Catheter tip positioning place at first attempt , n(%) |  | |  | | 0.0526 |
| SVC upper 1/3 | 24/276 (8.7%) | | 24/274 (8.8%) | |  |
| SVC middle 1/3 | 44/276 (15.9%) | | 41/274 (15.0%) | |  |
| SVC lower 1/3 | 87/276 (31.5%) | | 60/274 (21.9%) | |  |
| SVC/RA junction | 87/276 (31.5%) | | 93/274 (33.9%) | |  |
| RA upper 1/3 | 23/276 (8.3%) | | 28/274 (10.2%) | |  |
| RA middle 1/3 | 4/276 (1.4%) | | 8/274 (2.9%) | |  |
| RA lower 1/3 | 0/276 | | 2/274 (0.7%) | |  |
| Other places | 7/276 (2.5%) | | 18/274 (6.6%) | |  |
|  |  | |  | |  |
| First-attempt target rate , n(%) | 242/276 (87.7%) | | 218/274 (79.6%) | | 0.0101 |
| 95% CI | (83.8% to 91.6%) | | (74.8% to 84.3%) | |  |
| Percent difference (95% CI) vs. Landmark | 8.1% (1.6% to 14.6%) | | - | |  |
|  |  | |  | |  |
| Optimal target rate , n(%) | 174/276 (63.0%) | | 153/274 (55.8%) | | 0.0853 |
| 95% CI | (57.3% to 68.7%) | | (50.0% to 61.7%) | |  |
| Percent difference (95% CI) vs. Landmark | 7.2% (-1.3% to 15.8%) | | - | |  |
| Right atrium rate , n(%) | 27/276 (9.8%) | | 38/274 (13.9%) | | 0.1378 |
| 95% CI | (6.3% to 13.3%) | | (9.8% to 18.0%) | |  |
| Percent difference (95% CI) vs. Landmark | -4.1% (-9.8% to 1.7%) | | - | |  |

CI: Confidence Interval, IC ECG: Intracavitary Electrocardiograph, PICC: Peripherally Inserted Central Catheter, RA: Right Atrium, SVC: Superior Vena Cava.
