# Supplementary material for: Superior success rate of intracavitary electrocardiogram guidance for peripherally inserted central catheter placement in patients with cancer: A randomized open-label controlled multicenter study
Source: PLoS One. 2017 Mar 9;12(3):e0171630. doi: 10.1371/journal.pone.0171630 (PMC5344315; doi:10.1371/journal.pone.0171630)
Supplement: S4 Table — PICCs: peripherally inserted central catheters. (DOCX) [file pone.0171630.s008.docx]

**S4 Table: Catheter tip placement via first attempt by ultra-sound or chest x-ray confirmation and corresponding endpoint rates with IC ECG versus landmark technique in** p**atients with basilic vein used for PICCs (N=892)**

|  |  | **PICC Method** | |  |  |
| --- | --- | --- | --- | --- | --- |
|  | **IC ECG**  **(N=438)** | | **Landmark**  **(N=454)** | | **p-value** |
| Catheter tip positioning place at first attempt, n(%) |  | |  | | <0.0001 |
| SVC upper 1/3 | 38/437 (8.7%) | | 35/451 (7.8%) | |  |
| SVC middle 1/3 | 70/437 (16.0%) | | 64/451 (14.2%) | |  |
| SVC lower 1/3 | 145/437 (33.2%) | | 103/451 (22.8%) | |  |
| SVC/RA junction | 136/437 (31.1%) | | 148/451 (32.8%) | |  |
| RA upper 1/3 | 35/437 (8.0%) | | 56/451 (12.4%) | |  |
| RA middle 1/3 | 6/437 (1.4%) | | 18/451 (4.0%) | |  |
| RA lower 1/3 | 1/437 (0.2%) | | 2/451 (0.4%) | |  |
| Other places | 6/437 (1.4%) | | 25/451 (5.5%) | |  |
|  |  | |  | |  |
| First-attempt target rate , n(%) | 389/437 (89.0%) | | 350/451 (77.6%) | | <0.0001 |
| 95% CI | (86.1% to 91.9%) | | (73.8% to 81.5%) | |  |
| Percent difference (95% CI) vs. Landmark | 11.4% (6.3% to 16.5%) | | - | |  |
|  |  | |  | |  |
| Optimal target rate , n(%) | 281/437 (64.3%) | | 251/451 (55.7%) | | 0.0086 |
| 95% CI | (59.8% to 68.8%) | | (51.1% to 60.2%) | |  |
| Percent difference (95% CI) vs. Landmark | 8.6% (2.0% to 15.3%) | | - | |  |
| Right atrium rate , n(%) | 42/437 (9.6%) | | 76/451 (16.9%) | | 0.0015 |
| 95% CI | (6.8% to 12.4%) | | (13.4% to 20.3%) | |  |
| Percent difference (95% CI) vs. Landmark | -7.2% (-11.9% to -2.6%) | | - | |  |

CI: Confidence Interval, IC ECG: Intracavitary Electrocardiograph, PICC: Peripherally Inserted Central Catheter, RA: Right Atrium, SVC: Superior Vena Cava.
